# Anti-angiogenic efficacy of 5′-triphosphate siRNA combining VEGF silencing and RIG-I activation in NSCLCs

**DOI:** 10.18632/oncotarget.4869

**Published:** 2015-08-27

**Authors:** Dongmei Yuan, Mao Xia, Gang Meng, Chun Xu, Yong Song, Jiwu Wei

**Affiliations:** ^1^ Jiangsu Key Laboratory of Molecular Medicine, Medical School and the State Key Laboratory of Pharmaceutical Biotechnology, Nanjing University, Nanjing, China; ^2^ Department of Respiratory Medicine, Jinling Hospital, Nanjing, China; ^3^ Nanjing University Hightech Institute at Suzhou, Suzhou, China; ^4^ Drum Tower Hospital, Medical School of Nanjing University, Nanjing, China

**Keywords:** short interfering RNA, RIG-I, VEGF, NSCLC, anti-angiogenesis

## Abstract

Short interfering RNA (siRNA) targeting angiogenic factors and further inhibiting tumor angiogenesis, is one of the potent antitumor candidates for lung cancer treatment. However, this strategy must be combined with other therapeutics like chemotherapy. In this study, we designed a 5′-triphosphate siRNA targeting VEGF (ppp-VEGF), and showed that ppp-VEGF exerted three distinct antitumor effects: i) inhibition of tumor angiogenesis by silencing VEGF, ii) induction of innate immune responses by activating RIG-I signaling pathway, and thus activate antitumor immunity, iii) induction of apoptosis. In a subcutaneous model of murine lung cancer, ppp-VEGF displayed a potent antitumor effect. Our results provide a multifunctional antitumor molecule that may overcome the shortages of traditional antiangiogenic agents.

## INTRODUCTION

Lung cancer is the leading cause of cancer-related mortality in human, and non-small cell lung cancer (NSCLC) accounts for approximately 80% of all lung cancers [1]. Special network of angiogenesis, escape from immune destruction and resistance to cell death have been considered as hallmarks of cancer [2]. The concept of antiangiogenic therapy for cancer began with Folkman's balance hypothesis for the “angiogenic switch”. The prevailing evidence suggested that changes in the relative balance of inducers and inhibitors of angiogenesis can activate the “switch” [3]. In tumor tissue, the overexpressed angiogenic inducers trigger the “switch”, and promote formation of angiogenic network. Anti-angiogenic therapy targeting angiogenic network causes the insufficient regional blood supply of cancer, and eventually inhibits tumor growth.

Much attention has been focused on the vascular endothelial growth factor (VEGF) family and receptor tyrosin kinases that mediate pro-angiogenic effects [4]. The VEGF family includes VEGF-A, VEGF-B, VEGF-C, VEGF-D, and placental growth factor. The major mediator of tumor angiogenesis is VEGF-A, also referred to VEGF. VEGF has been shown to correlate with the invasion and metastasis of different types of cancer [5–6]. Meanwhile, VEGF in tumor microenvironment recruits endothelial progenitor cells to the tumor region and promotes angiogenesis of cancer [7]. Targeting VEGF combined with conventional chemotherapy has been investigated in advanced NSCLC patients and achieved an increased progression free survival in the first-line treatment [8–9]. However, VEGF inhibitors (such as bevacizumab) that can cause anti-angiogenesis should be used in combination with other therapies like chemotherapy instead of being used alone in cancer therapy [10].

Insufficient antitumor immunity is a critical hurdle for efficient cancer therapy. Provoking antitumor immune responses in patients holds promising for cancer therapy. It has been demonstrated that RNA with a triphosphate group at the 5′ end (ppp-RNA) is a specific ligand of retinoic-inducible gene I (RIG-I), a cytosolic pattern recognition receptor [11]. RIG-I has a DExD/H-box RNA helicase domain, a C-terminal domain (CTD), and a N-terminal caspase recruitment domain (CARD). Upon the recognition of viral ppp-RNA by CTD, ATP-dependent conformational change allows CARDs to interact with a downstream adaptor molecule [12]. RIG-I signaling pathway mediates innate immune responses characterized by the production of type I IFN [13–14]. Several preclinical studies have further confirmed that ppp-siRNA can robustly elicit type I IFN-mediated antitumor immune responses [15–17].

Moreover, ppp-RNAs have also been shown to trigger proapoptotic signaling predominantly in tumor cells, while nonmalignant cells are protected from proapoptotic signaling via Bcl-x_L_ [18]. A more recent study shows that RIG-I-like helicase also triggers immunogenic cell death in pancreatic cancer cells [16]. These results suggest that ppp-RNA can also be used to overcome cell death resistance.

Short interfering RNAs (siRNAs) can be designed to target mRNA encoding key regulators for tumor survival with high specificity [19]. SiRNA directed against VEGF and kinesin spindle protein (KSP) has been shown both cleavage activity of mRNA and antitumor activity in phase I clinical trial [20]. However, due to the limitation of VEGF-siRNA with sole silencing function, we postulate that ppp-RNA orchestrating RIG-I activation to VEGF-siRNA (ppp-VEGF), should be a good solution and a novel strategy for cancer therapy. In this study, we designed a multifunctional ppp-VEGF and investigated its potential antitumor activities, i.e., inhibition of tumor neovascularization, immune activation and proapoptotic efficacy in NSCLCs.

## RESULTS

### ppp-VEGF efficiently silences VEGF expression in NSCLC cells

**Figure 1:**
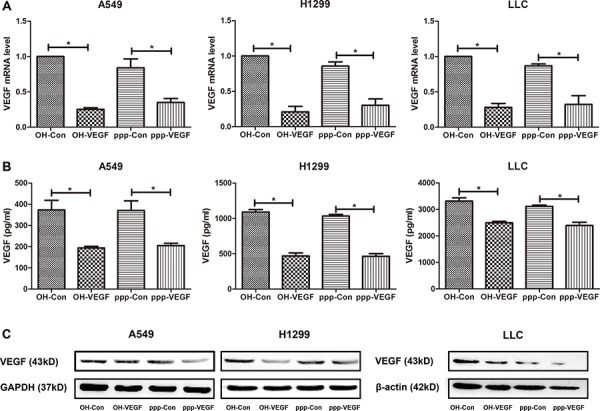
ppp-VEGF efficiently silences VEGF expression in NSCLC cells Various lung cancer cell lines A549, H1299 and LLC were treated with nonspecific siRNA (OH-Con, ppp-Con), or siRNA targeting VEGF (OH-VEGF, ppp-VEGF) for 48 hours. **A.** Total mRNA was then isolated and VEGF mRNA expression was analyzed by qRT-PCR. Or **B.** supernatants were harvested and VEGF secretion was determined by ELISA. Or **C.** cell lysates were harvested and VEGF protein level was determined by western blot. Similar results were obtained in two independent experiments.*p < 0.05.

To combine RIG-I activation with RNAi-mediated silencing of VEGF in one RNA molecule, we designed a siRNA targeting both human and murine VEGF, and generated a 5′-triphosphate-VEGF-specific siRNA (ppp-VEGF) by *in vitro* transcription using a DNA template with the same sequence containing the T7 RNA polymerase promoter sequence [11]. Then we tested the gene silencing efficiency in various lung cancer cell lines. As shown in Figure 1, ppp-VEGF efficiently silenced VEGF expression on mRNA level (Figure 1A) and reduced the protein production (Figure 1B & 1C). Thus, ppp-VEGF possesses the specific gene silencing function similar as the conventional siRNA targeting VEGF (OH-VEGF).

### ppp-VEGF inhibits angiogenesis in tumor-bearing mice, impairs tubule-like structure formation, and decreases migration of endothelial cells *in vitro*

**Figure 2:**
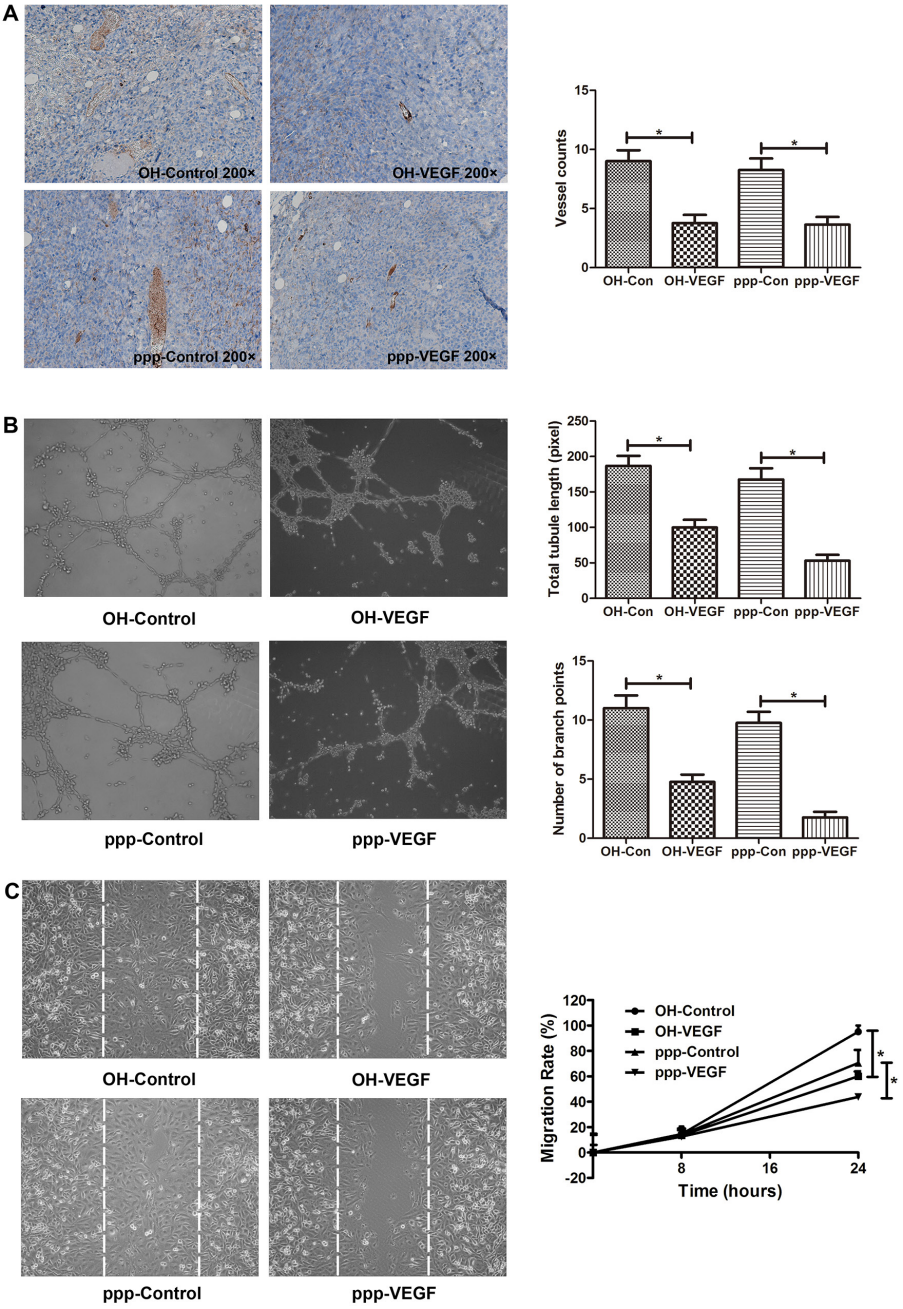
ppp-VEGF inhibits tumor angiogenesis *in vivo*, inhibits tubule-like structure formation, and decreases migration of endothelial cells *in vitro* **A.** LLC cells were subcutaneously implanted into C57BL/6 mice. When the tumors reached to about 0.5 cm in diameter, mice received intratumoral injection of OH-control, OH-VEGF, ppp-control or ppp-VEGF. The dose of siRNA was 20 μg for each injection and twice a week for 2 weeks. Then tumor samples were obtained and subjected to immunohistochemical staining against CD31. Tumor vessels were analyzed and counted. **B.** Human endothelial cell line ECV304 cells were seeded onto Matrigel-coated 24-well plates and then treated with OH-control, OH-VEGF, ppp-control and ppp-VEGF, respectively. Total tubule length was measured only for the tubular structures connecting 2 cell clusters in high-power microscopy. The number of branching points was used as another parameter to quantify the tubule formation. Means of triplicates are shown. Similar results were obtained in two independent experiments. **C.** ECV304 cells were seeded onto 6-well plates (2 × 10^5^ cells per well) for 24 h and then scratches were made with a 200 μl sterile pipette tip. Cells were then treated with OH-control, OH-VEGF, ppp-control, ppp-VEGF, respectively. The distance between the wound boundaries was dynamically monitored and photographed with an inverted phase-contrast microscope (left panel), white dotted lines depict the borderlines at 0 hour. The migration rate was then counted (right panel). *p < 0.05.

Having shown the specific VEGF inhibitory effect of ppp-VEGF in cancer cells, we next evaluated the antiangiogenic efficacy of ppp-VEGF in C57BL/6 mice bearing subcutaneous lung cancers. By intratumoral injection, ppp-VEGF showed potent angiogenic inhibitory efficacy (Figure 2A). Furthermore, we wanted to know if ppp-VEGF had direct influence on non-malignant endothelial cells, which play crucial roles in tumor neovascularization. Indeed, using *in vitro* tubule formation assay, we found that ppp-VEGF treated endothelial cells failed to form intact tubules, as evidenced by the reduced tubule length and branching points (Figure 2B). In line, the scratch wound migration assay demonstrated that ppp-VEGF decreased the migration of endothelial cells (Figure 2C). These results confirm that ppp-VEGF efficiently inhibits angiogenesis.

### ppp-VEGF activates RIG-I signaling and induces type I IFN responses *in vitro*

**Figure 3:**
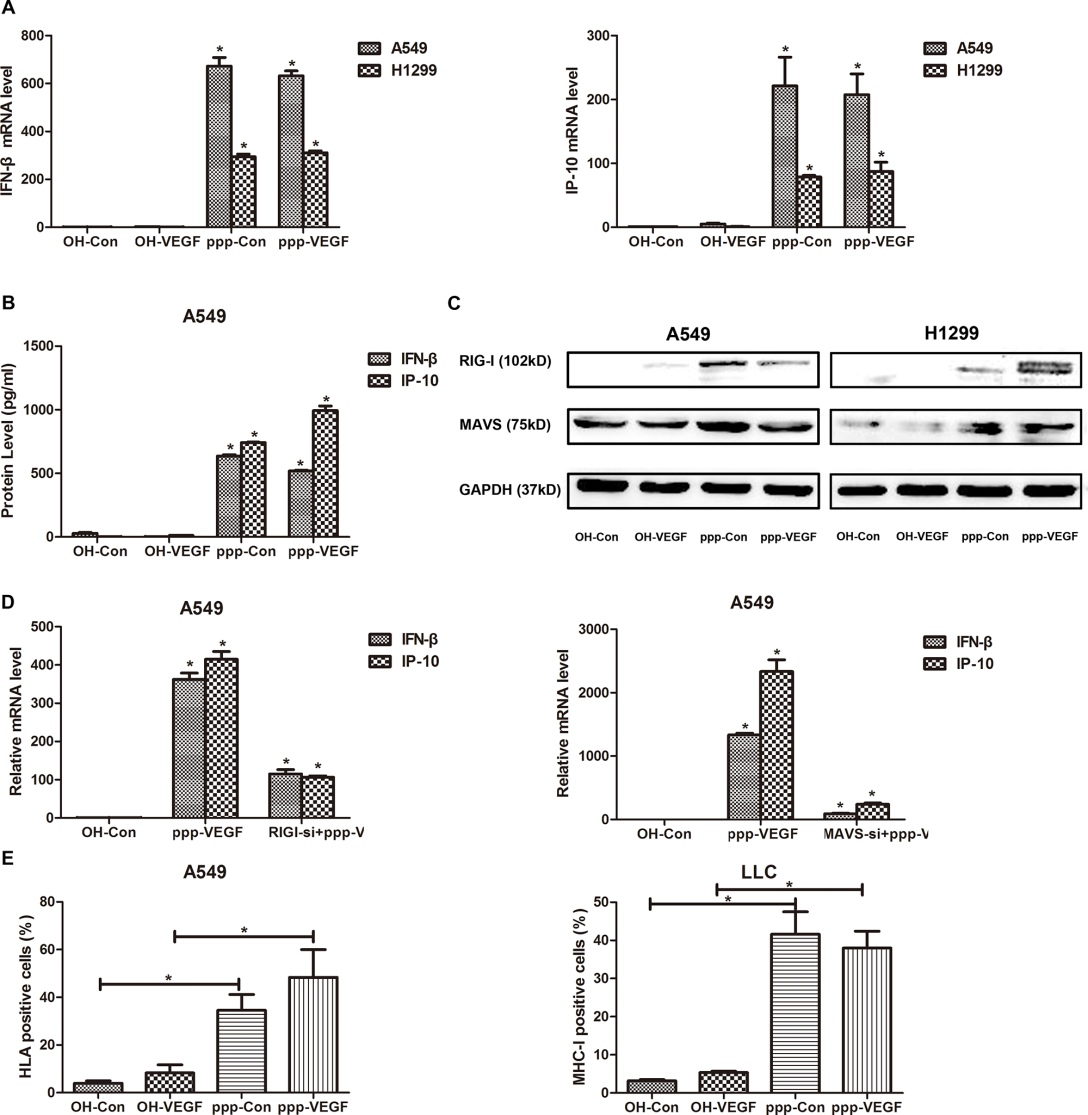
ppp-VEGF activates RIG-I signaling and induces type I IFN responses *in vitro* A549 and H1299 were treated with OH-control, OH-VEGF, ppp-control or ppp-VEGF for 48 h, then **A.** total mRNAs were harvested, IFN-β mRNA expression and downstream IP-10 mRNA expression were quantified by qRT-PCR, or **B.** the supernatant of A549 cells was collected and production of IFN-β and IP-10 was quantified by Elisa, or **C.** cell lysates of A549 and H1299 cells were harvested, the expression of RIG-I and MAVS was assessed by western blot. **D.** A549 cells were transfected with nonspecific control siRNA or specific siRNA targeting RIG-I for 24 hours, and then cells were treated with OH-Control or ppp-VEGF. The expression of IFN-β and IP-10 was detected by qRT-PCR. **E.** A549 and LLC cells were treated with OH-control, OH-VEGF, ppp-control or ppp-VEGF for 48 h, the expression of HLA or MHC-I was determined by flow cytometry. Similar results were obtained in 2 independent experiments. *p < 0.05.

As we have shown in previous study that ppp-RNA induces antitumor immunity [16], we sought to confirm if ppp-VEGF could also activate RIG-I signaling in NSCLCs. After ppp-VEGF treatment, mRNA expression of type I interferon beta (IFN-β), and its downstream target IP-10, was robustly increased in lung cancer cells (Figure 3A). Accordingly, secretion of IFN-β and IP-10 was also massively increased in ppp-VEGF treated cancer cells (Figure 3B). Moreover, we confirmed that ppp-RNA induced innate immune response was mediated by RIG-I/ mitochondrial associated viral sensor (MAVS) signaling pathway, as both RIG-I and MAVS were up-regulated upon ppp-RNA treatment (Figure 3C), while the up-regulation of IFN-β and IP-10 induced by ppp-VEGF was significantly blocked in cells with RIG-I or MAVS silencing (Figure 3D). Finally, we found that ppp-VEGF markedly up-regulated the expression of major histocompatibility complex class I (MHC-I) on cancer cells (Figure 3E), which may render tumor cells more susceptible to immune attack. Together, these results suggest that ppp-VEGF efficiently induces innate immune responses via activation of RIG-I signaling pathway, and thus may enhance antitumor immunity.

### ppp-VEGF induces apoptosis in NSCLC cells

**Figure 4:**
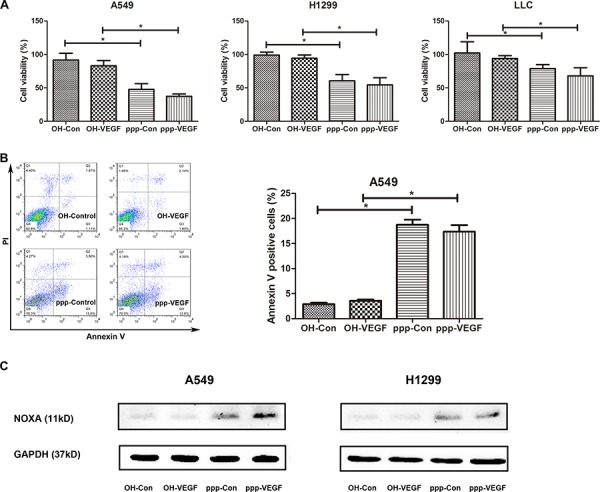
ppp-VEGF induces apoptosis in NSCLC cells A549, H1299, LLC cells were treated with OH-control, OH-VEGF, ppp-control or ppp-VEGF for 48 h, **A.** cell viability was then detected by MTT assay, or **B.** A549 cells were stained with Annexin V/propidium iodide and subjected to flow cytometry. Similar results were obtained in 2 independent experiments. **C.** The expression of NOXA was assessed by western blot in A549 and H1299 cells. *p < 0.05.

As we and others have shown before that ppp-RNA induces apoptosis in cancer cells [16, 18], we then further investigated whether ppp-VEGF also induces apoptosis in lung cancer cells. Indeed, ppp-VEGF exerted significant antitumor activity in NSCLCs, which was evidenced by the decreased cell viability (Figure 4A), and increased annexin V positive cells (Figure 4B). It has been reported that RIG-I activation can stimulate the mitochondrial apoptotic pathway in a BH3-only protein NOXA-dependent pathway in melanoma cells [18]. In agreement with this, we found that ppp-siRNAs also triggered NOXA upregulation in lung cancer cells (Figure 4C). Taken together, these results suggest that lung cancer cells are sensitive to ppp-VEGF induced apoptosis.

### ppp-VEGF exerted potent antitumor efficacy in tumor-bearing mice

**Figure 5:**
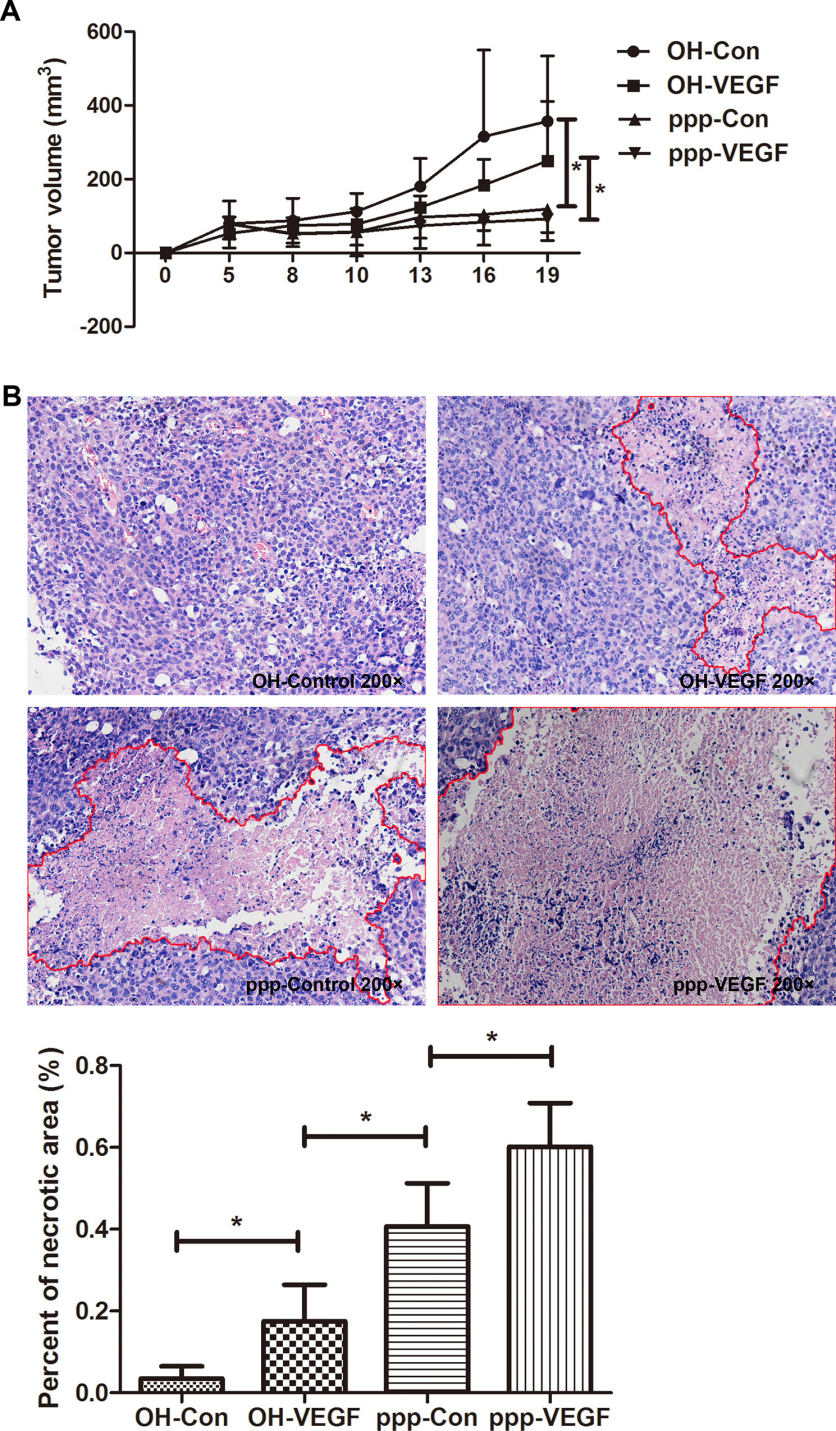
ppp-VEGF inhibits tumor growth and significantly promotes tumor necrosis C57BL/6 mice were injected subcutaneously with LLC cells. When tumor diameters reached to 0.5 cm, mice were randomized to 4 groups (*n* = 5 each group), and received intratumoral injection of OH-control, OH-VEGF, ppp-control and ppp-VEGF, respectively. 20 μg siRNA coupled in Lipofectamine^2000^ was used for each injection, twice a week for two weeks. **A.** The tumor volumes were measured on days as indicated. **B.** The mice were sacrificed on day 19 and tumor specimens were analyzed by H&E staining, tumor necrotic areas were depicted by red lines (upper panel). Percent of necrotic area to total tumor area of each was measured. Means + SD of each group are shown (lower panel). *p < 0.05.

Finally, we evaluated the therapeutic efficacy of ppp-VEGF *in vivo*. We found that ppp-Control and ppp-VEGF significantly inhibited tumor growth (Figure 5A) by triggering massive tumor cell death (Figure 5B). Interestingly, we found that average necrotic area in ppp-VEGF treated tumors was significantly higher than that treated with ppp-control (Figure 5B), indicating that inhibition of tumor vascularization also contributed to antitumor effect. No obvious toxicity was observed in ppp-VEGF and other experimental groups. Taken together, these results indicate that ppp-VEGF possesses potent antitumor activity.

## DISCUSSION

Neovascularization, immune escape and cell death resistance are key hallmarks of cancer [2]. Endeavors have been made to overcome these in cancer therapy. In this study, we showed that a single molecule ppp-VEGF, exerted three distinct antitumor activities, angiogenic inhibition, immune activation and cell death induction, which led to enhanced antitumor efficacy in NSCLC.

Angiogenesis is a complex process that is essential for cancer progression. VEGF is one of the most potent mediators in promoting tumor angiogenesis. Elevated VEGF level is correlated with a worse prognosis in patients with lung cancer [21]. It has been demonstrated that selective inhibition of VEGF binding to VEGFR2 with a fully humanized monoclonal antibody leads to reduced tumor angiogenesis [22]. To update, most of the anti-angiogenic agents cannot be used alone and have to be combined with other therapeutics, such as chemotherapy, targeted therapy [23]. Furthermore, it has been reported that anti-angiogenic therapy transiently normalizes abnormal tumor vasculature, it also reduces in parallel both perfusion and net influx rate of docetaxel into tumor burdens [24]. It raises the debate that anti-angiogenic therapy in combination with chemotherapy might not be an optimal strategy for lung cancer treatment. In this study, the single molecule ppp-VEGF coordinately inhibited the VEGF-mediated tumor angiogenesis, activated the RIG-I-mediated immune responses and induced tumor cell apoptosis, which cleverly circumvented the disadvantages of single VEGF targeting or the combination. Moreover, we found that ppp-VEGF not only sufficiently reduced VEGF production and secretion in lung cancer cells, but also directly disrupted migration and tubular formation of endothelial cells, which plays central role in angiogenesis. This makes ppp-VEGF extremely attractive as a candidate for anti-angiogenic cancer therapy.

Our previous study has shown that ppp-RNA effectively induces type I IFNs production in tumor cells and thus activates antitumor immunity *in vivo* [16]. In line, we also confirmed that ppp-VEGF massively induced type I IFN production, and upregulated MHC-I expression in lung cancer cells. It has been reported that blocking VEGF activity in solid tumors extends beyond inhibition of angiogenesis. For instance, anti-VEGF therapy may modulate immune cell infiltration, as well as the intra-tumoral and serum cytokine levels in preclinical models of breast cancer [25], suggesting that anti-VEGF therapies might function as modulators of antitumor immunity. Thus, we postulate that ppp-VEGF may be a promising inducer of antitumor immunity by combining RIG-I activation to VEGF silencing.

In agreement with previous studies, ppp-VEGF also effectively triggered tumor cell death. Since RIG-I activation can stimulate the mitochondrial apoptotic pathway in a BH3-only protein NOXA-dependent but p53-independent manner [18], it is reasonable that ppp-RNA may also work well against cancer cells with p53 mutations, including NSCLC [26]. Interestingly, we found that tumor volumes in mice treated with ppp-VEGF were comparable to that treated with ppp-control, however, the average necrotic area within tumor mass obtained from ppp-VEGF-treated mice was significantly increased. The plausible explanation for this observation is that ppp-VEGF induced massive liquefactive necrosis within tumor burdens and thus, tumor volumes might not exhibit significant reduction. The increased necrosis could be due to the orchestrated effect of apoptosis triggered by ppp-RNA, and insufficient blood supply induced by VEGF-targeting.

Another critical issue is the tumor microenvironment involving pro- and anti-angiogenic pathways. For instance, the reduced infiltration of tumors with cancer-associated fibroblasts was correlated with the efficacy of VEGF-targeting drugs [27]. The cross-talk between TGF-β and VEGF signaling pathways was involved in complex pro- and anti-angiogenesis in glioblastoma [28]. The complex *in vivo* microenvironment should be taken into consideration before clinical translation of ppp-VEGF.

In conclusion, ppp-VEGF combines anti-angiogenesis, immune activation and cell death induction in a single molecule, which can be further developed to a potent therapeutic antitumor drug against lung cancer. This molecule might be also suitable for other types of cancer.

## MATERIALS AND METHODS

### Cell lines

Human lung adenocarcinoma cell lines A549, H1299, and murine lung cancer cell line LLC were used in this research. Cells were cultured in Dulbecco's Modified Eagle Medium (DMEM) supplemented with 5% fetal bovine serum (FBS) (Gibco), 2 mM L-glutamine and 100 U/l penicillin and 0.1 mg/ml streptomycin (all from Invitrogen). Human endothelial cell line ECV304 cells were grown in RPMI-1640 (Gibco) medium containing 10% FBS, 100 μg/ml penicillin/streptomycin. And all cell lines were incubated in a humidified incubator with 5% CO_2_ at 37°C.

### siRNA and ppp-siRNA

The siRNA against both human and murine VEGF (OH-VEGF) was constructed according to a previous study containing a two deoxythymidine overhang at the 3′ end [29]. The sequences were: forward 5′-AUGUGAAUGCAGACCAAAGAA-3′ and reverse 5′-UUCUUUGGUCUGCAUUCACAU-3′. The sequences of nonspecific siRNA (OH-control) were: forward 5′-UUCUCCGAACGUGUCACGU-3′ and reverse 5′-ACGUGACACGUUCGGAGAA-3′. These siRNAs were synthesized by Invitrogen (China). The matching 5′-triphosphate-modified siRNA was transcribed using the correlating DNA template that contained the T7 RNA polymerase promoter sequence. *In vitro* transcription and purification of ppp-RNA was done using the Megashort SkriptTM T7 Kit (ABI, AM1354) according to the manufacturer's instruction. SiRNAs targeting RIG-I and MAVS were also purchased from Invitrogen (China). The sequences were as follows. RIG-I (HSS177513): forward 5′-AUCACGGAUUAGCGACAAA-3′ and reverse 5′-UUUGUCGCUAAUCCGUGAU-3′, MAVS (HSS148538): forward 5′-UCGCAACUCUACUU CUGCCGCUAUU-3′ and reverse 5′-AAUAGCGGCA GAAGUAGAGUUGCGA-3′.

### siRNA transfection

For *in vitro* experiments, siRNAs in OptiMEN (Gibco) were mixed with Lipofectamine^2000^ reagent (Invitrogen, 11668-019) at a concentration of 1 μg/ml. Then the mixture was added into cells. For *in vivo* administration of siRNAs, 20 μg siRNA was coupled by 10 μl Lipofectamine^2000^ in a final volume of 20 μl for intratumoral injection.

### mRNA extraction and qRT-PCR

Total RNA was extracted from cells using the TRIzol reagent (Invitrogen, USA, 15596–026) following the manufacturer's recommendations. RNA was reverse transcribed (TaKaRa, Shiga, Japan, DRR036A). qPCR was performed using the Real-Time PCR system (ABI 7300, Advanced Biosystems, Foster, CA). Gene expression was calculated with the comparative Ct method and normalized to the endogenous levels of GAPDH or β-actin. Primer sequences used for qRT-PCR are as follows: Human GAPDH, forward 5′-CCATGTTCGTCATGGGTGTGAAC-3′ and reverse 5′-GCCAGTAGAGGCAGGGATGATGTTC-3′. Murine β-actin, forward 5′-AACAGTCCGCCTAGAAGCAC-3′ and reverse 5′-CGTTGACATCCGTAAAGACC-3′. Human VEGF, forward 5′-GCACCCATGGCAG AAGGAGG-3′ and reverse 5′-CCTTGGTGAGGTTT GATCCGCATA-3′. Murine VEGF, forward 5′-GGA CCCTGGCTTTACTGC-3′ and reverse 5′-CGGGCTT GGCGATTTAG-3′. Human IP-10, forward 5′-CTTCCAA GGATGGACCACACA-3′ and reverse 5′-CCTTCCTA CAGGAGTAGTAGCAG-3′. Human IFN-β, forward 5′-CTTGGATTCCTACAAAGAAGC-3′ and reverse 5′-CATCTCATAGATGGTCAATGC-3′.

### ELISA

Cytokine levels in cell culture supernatant were quantified by ELISA for IP-10 (eBiosciences, BMS284), IFN-β (PBL Interferon source, 41410–1A), and VEGF (Human; eBiosciences, BMS277; Mouse; eBiosciences, BMS619). All assays were duplicated and performed according to the manufacturer's instruction.

### Cell viability assay

The cell viability of lung cancer cells was detected using the MTT assay (Sigma) in 96-well plates at a cell density of 3 × 10^3^ per well. In the late period of incubation, 20 μl of a stock solution of MTT was added to each well for 3 h. Then cells were lysed with dimethyl sulfoxide and quantified at OD570 using an ELISA reader.

### Flow cytometry

MHC-I expression on human or murine lung cancer cells was determined using anti-human HLA-ABC-FITC (eBioscience, San Diego, CA, 11–9983) or anti-mouse H-2K^b^-FITC (eBiosciences, 11-5958) antibody for 30 min at 4°C. Cells were then washed twice with phosphate buffered saline (PBS) and subjected to flow cytometry (Becton, Dickinson and Company, Franklin Lakes, NJ). The mouse IgG2a Kappa isotype control FITC antibody (eBioscience, 11–4724) was used as control.

Apoptotic cell death was determined by staining with Annexin V/propidium iodide (PI) (Invitrogen, V13241). Cells were harvested and washed once with PBS, then resuspended in 100 μl binding buffer followed by incubation with 2.5 μl Annexin V per test for 20 min. Then, 1 μl PI per test was added and stained cells were subjected to flow cytometry.

### Western blot

Cells were homogenized in cell lysis buffer (Beyotime), and protein concentration was determined using a BCA kit (Beyotime). Equal amounts of protein were separated by sodium dodecyl sulfate-polyacrylamide gel electrophoresis (SDS-PAGE) and electrophoretically transferred onto a polyvinylidene fluoride (PVDF) membrane (Roche, 03010040001). After blocking with 5% nonfat milk in Tris-buffered saline containing 0.1% Tween-20, the membrane was incubated with specific primary antibodies. Then the membrane was incubated with appropriate HRP-conjugated secondary antibodies. Signals were monitored by an enhanced chemiluminescence reagent (Millipore, WBKLS0500) and subjected to Alpha Innotech Flour Chem-FC2 imaging system (Alpha Innotech). The antibodies used in this study are as follows: VEGF (abcam, ab46154), RIG-I (Cell signaling technology, 3743), MAVS (abcam, ab31334), NOXA (abcam, ab13654), GAPDH (Bioworld Technology, MB001), β-actin (Cell signaling technology, 4967).

### *In vitro* tubule formation assay

5 × 10^4^ ECV304 cells were seeded in onto Matrigel coated 24-well plates and then treated with various siRNAs. Tubule-like structures were monitored under upright microscope. Total tube length was measured only for the tubular structures connecting 2 cell clusters in high-power field. The number of branching points was used as another parameter to quantify tube formation.

### Scratch assay

ECV304 cells were seed in 6-well tissue culture plates (2 × 10^5^ cells per well). 24 hours later, scratches were made with a 200 μl sterile pipette tip. The cells were washed with PBS three times, and then transfected with siRNAs. The distance between wound boundaries was monitored and photographed with an inverted phase-contrast microscope, and then calculated at 0, 8, and 24 h after scratches. The migration rate was defined as (distance at 0 h – distance at 24 h) / distance at 0 h.

### Mice, tumor engraftment and therapy

6–8 week-old male C57BL/6 mice were obtained from Model Animal Research Center, Nanjing University, China. Mice were injected subcutaneously with 2 × 10^6^ LLC cells in the right flanks. When tumor size reached to about 0.5 cm in diameter, mice were then randomized to 4 groups (5 mice per group) followed by intratumoral injection of siRNAs (20 μg each injection). The mice received injections twice a week for two weeks. Tumors were measured and tumor volume was calculated as length × width^2^/2. Mice exhibiting moribund behavior were euthanized. All animal work was approved by the Animal Care Committee of Nanjing University in accordance with Institutional Animal Care and Use Committee guidelines.

### Immunohistochemical (IHC) staining

All tumor samples were fixed in 10% formalin, embedded in paraffin and cut to 5 μm-thick sections. The sections were deparaffinized and rehydrated. Slides were either stained by hemoatoxylin and eosin (H&E) for histological analysis and evaluation of average percentage of necrotic area within tumors, or manipulated for antigen retrieve before incubated with anti-mouse CD31 antibody (Abcam, ab28364). Peroxidase-conjugated secondary antibody was used to detect CD31. The CD31 positive stained vessels were counted. The mean number of vessels was defined as micro blood vessel density (MVD) [30].

### Statistical analysis

Significant differences were analyzed using two-tailed Student's *t*-test. Multiple comparisons were analyzed by on-way-ANOVA followed by Bonferroni posttest. All data were presented as mean ± standard error of the mean. Statistical analysis was performed using GraphPad Prism5 software and *p*-values less than 0.05 were considered significant.

## References

[1] Siegel R, Naishadham D, Jemal A (2013). Cancer Statistics. CA Cancer J Clin.

[2] Hanahan D, Wenberg RA (2011). Hallmarks of cancer: the next generation. Cell.

[3] Hanahan D, Fokman J (1996). Patterns and emerging mechanisms of the angiogenic switch during tumorigenesis. Cell.

[4] Kerbel RS (2008). Tumor angiogenesis. N Engl J Med.

[5] Jantus-Lewintre E, Sanmartin E, Sirera R, Blasco A, Sanchez JJ, Taron M, Rosell R, Camps C (2011). Combined VEGF-A and VEGFR-2 concentrations in plasma: diagnostic and prognostic implications in patients with advanced NSCLC. Lung Cancer.

[6] Inoue M, Hager JH, Ferrara N, Gerber HP, Hanahan D (2002). VEGF-A has a critical, nonredundant role in angiogenic switching and pancreatic beta cell carcinogenesis. Cancer Cell.

[7] Wei J, Blum S, Unger M, Jarmy G, Lamparter M, Geishauser A, Vlastos GA, Chan G, Fischer KD, Rattat D, Debatin KM, Hatzopoulos AK, Beltinger C (2004). Embryonic endothelial progenitor cells armed with a suicide gene target hypoxic lung metastases after intravenous delivery. Cancer Cell.

[8] Cabebe E, Wakelee H (2007). Role of anti-angiogenesis agents in treating NSCLC: focus on bevacizumab and VEGFR tyrosin kinase inhibitors. Curr Treat Options Oncol.

[9] Reck M, von Pawel J, Zatloukal P, Ramlau R, Gorbounova V, Hirsh V, Leighl N, Mezger J, Archer V, Moore N, Manegold C (2009). Phase III trial of cisplatin plus gemcitabine with either placebo or bevacizumab as first-line therapy for nonsquamous non-small-cell lung cancer: AVAiL. J Clin Oncol.

[10] Hurwitz H, Fehrenbacher L, Novotny W, Cartwright T, Hainsworth J, Heim W, Berlin J, Baron A, Griffing S, Holmgren E, Ferrara N, Fyfe G, Rogers B (2004). Bevacizumab plus irinotecan, fluorouracil, and leucovorin for metastatic colorectal cancer. N Eng J Med.

[11] Hornung V, Ellegast J, Kim S, Brzozka K, Jung A, Kato H, Poeck H, Akira S, Conzelmann KK, Schlee M, Endres S, Hartmann G (2006). 5′-Triphosphate RNA is the ligand for RIG-I. Science.

[12] Yoneyama M, Onomoto K, Joqi M, Akaboshi T, Fujita T (2015). Viral RNA detection by RIG-I-like receptors. Curr Opin Immunol.

[13] Yoneyama M, Fujita T (2009). RNA recognition and signal transduction by RIG-I-like receptors. Immunol Rev.

[14] Chen X, Qian Y, Yan F, Tu J, Yang X, Xing Y, Chen Z (2013). 5′-Triphosphate-siRNA activates RIG-I-dependent type I interferon production and enhances inhibition of hepatitis B virus replicaion in HepG2.2.15 cells. Eur J Pharmacol.

[15] Poeck H, Besch R, Maihoefer C, Renn M, Tormo D, Morskaya SS, Kirschnek S, Gaffal E, Landsberg J, Hellmuth J, Schmidt A, Anz D, Bscheider M (2008). 5′-Triphosphate-siRNA: turning gene silencing and Rig-I activation against melanoma. Nat Med.

[16] Ellermeier J, Wei J, Duewell P, Hoves S, Stieg MR, Adunka T, Noerenberg D, Anders HJ, Mayr D, Poeck H, Hartmann G, Endres S, Schnurr M (2013). Therapeutic efficacy of bifunctional siRNA combining TGF-β1 silencing with RIG-I activation in pancreatic cancer. Cancer Res.

[17] Meng G, Xia M, Xu Chun, Yuan D, Schnurr M, Wei J (2013). Multifunctional antitumor molecule 5′-triphaosphate siRNA combining glutaminase silencing and RIG-I activation. Int J Cancer.

[18] Besch R, Poeck H, Hohenauer T, Senft D, Hacker G, Berking C, Hornung V, Endres S, Ruzicka T, Rothenfusser S, Hartmann G (2009). Proapoptotic signaling induced by RIG-I and MDA-5 results in type I interferon-independent apoptosis in human melanoma cells. J Clin Invest.

[19] McManus MT, Sharp PA (2002). Gene silencing in mammals by small interfering RNAs. Nat Rev Genet.

[20] Tabernero J, Shapiro GI, LoRusso PM, Cervantes A, Schwartz GK, Weiss GJ, Paz-Ares L, Cho DC, Infante JR, Alsina M, Gounder MM, Falzone R, Harrop J (2013). First-in-humans trial of an RNA interference therapeutic targeting VEGF and KSP in cancer patients with liver involvement. Cancer Discov.

[21] Carrillo de Santa Pau E, Arias FC, Caso Pelaez E, Munoz Molina GM, Sanchez Hernandez I, Muguruza Trueba I, Moreno Balsalobre R, Sacristan Lopez S, Gomez Pinillos A, del Val Toledo Lobo M (2009). Prognostic significance of the expression of vascular endothelial growth factors A, B, C, and D and their receptors R1, R2, and R3 in patients with nonsmall cell lung cancer. Cancer.

[22] Sandler A, Gray R, Perry MC, Brahmer J, Schiller JH, Dowlati A, Lilenbaum R, Johnson DH (2006). Paclitaxel-carboplatin alone or with bevacizumab for non-small-cell lung cancer. N Engl J Med.

[23] Falchook GS, Naing A, Hong DS, Zinner R, Fu S, Piha-Paul SA, Tsimberidou AM, Morgan-Linnell SK, Jiang Y, Bastida C, Wheler JJ, Kurzrock R (2013). Dual EGFR inhibition in combination with anti-VEGF treatement: a phase I clinical trial in non-small cell lung cancer. Oncotarget.

[24] Van der Veldt AA, Lubberink M, Bahce I, Walraven M, de Boer MP, Greuter HN, Hendrikse NH, Eriksson J, Windhorst AD, Postmus PE, Verheul PE, Serne EH, Lammertsma AA (2012). Rapid decrease in delivery of chemotherapy to tumors after anti-VEGF therapy: implications for scheduling of anti-angiogenic drugs. Cancer Cell.

[25] Roland CL, Lynn KD, Toombs JE, Dineen SP, Udugamasooriya DG, Brekken RA (2009). Cytokine levels correlate with immune cell infiltration after anti-VEGF therapy in preclinical mouse models of breast cancer. PLoS One.

[26] Andjelkovic T, Bankovic J, Stojsic J, Milinkovic V, Podolski-Renic A, Ruzdijic S, Tanic N (2011). Coalterations of p53 and PTEN tumor suppressor genes in non-small cell lung carcinoma patients. Transl Res.

[27] Ben-Batalla I, Cubas-Cordova M, Udonta F, Wroblewski M, Waizenegger JS, Janning M, Sawall S, Gensch V, Zhao L, Martinez-Zubiaurre I, Riecken K, Fehse B, Pantel K (2015). Cyclooxygenase-2 blockade can improve efficacy of VEGF-targeting drugs. Oncotarget.

[28] Krishnan S, Szabo E, Burghardt I, Frei K, Tabatabai G, Weller M (2015). Modulation of cerebral endothelial cell function by TGF-β in glioblastoma: VEGF-dependent angiogenesis versus endothelial mesenchymal transition. Oncotarget.

[29] Filleur S, Courtin A, Ait Si Ali S, Guglielmi J, Merle C, Harel Bellan A, Clezardin P, Cabon F (2003). SiRNA mediated inhibition of vascular endothlieal growth factor severely limits tumor resistence to antiangiogenic thrombospondin-1 and vascularization and growth. Cancer Res.

[30] Mohammed RA, Green A, EI-Shikh S, Paish EC, Ellis IO, Martin SG (2007). Prognostic significance of vascular endothelial growth factors-A, -C, and -D in breast cancer and their relationship with angio- and lymphangiogenesis. Br J Cancer.

